# Urolithin A Is a Dietary Microbiota-Derived Human Aryl Hydrocarbon Receptor Antagonist

**DOI:** 10.3390/metabo8040086

**Published:** 2018-11-29

**Authors:** Gulsum E. Muku, Iain A. Murray, Juan C. Espín, Gary H. Perdew

**Affiliations:** 1 Center for Molecular Toxicology and Carcinogenesis, Department of Veterinary and Biomedical Sciences, The Pennsylvania State University, University Park, PA 16802, USA; gem186@psu.edu (G.E.M.); iam1@psu.edu (I.A.M.); 2 The Huck Institutes of the Life Sciences, The Pennsylvania State University, University Park, PA 16802, USA; 3 Laboratory of Food & Health, Research Group on Quality, Safety and Bioactivity of Plant Foods, Department of Food Science and Technology, CEBAS-CSIC, 30100 Murcia, Spain; jcespin@cebas.csic.es

**Keywords:** aryl hydrocarbon receptor, polyphenols, inflammation, urolithin, AHR antagonist

## Abstract

Urolithins (e.g., UroA and B) are gut microbiota-derived metabolites of the natural polyphenol ellagic acid. Urolithins are associated with various health benefits, including attenuation of inflammatory signaling, anti-cancer effects and repression of lipid accumulation. The molecular mechanisms underlying the beneficial effects of urolithins remain unclear. We hypothesize that some of the human health benefits of urolithins are mediated through the aryl hydrocarbon receptor (AHR). Utilizing a cell-based reporter system, we tested urolithins for the capacity to modulate AHR activity. Cytochrome P450 1A1 (*CYP1A1*) mRNA levels were assessed by real-time quantitative polymerase chain reaction. Competitive ligand binding assays were performed to determine whether UroA is a direct ligand for the AHR. Subcellular AHR protein levels were examined utilizing immunoblotting analysis. AHR expression was repressed in Caco-2 cells by siRNA transfection to investigate AHR-dependency. UroA and B were able to antagonize 2,3,7,8-tetrachlorodibenzo-*p*-dioxin (TCDD)-induced AHR-mediated transcriptional activity. Furthermore, UroA and B attenuated TCDD-mediated stimulation of *CYP1A1* mRNA levels. In addition, competitive ligand binding assays characterized UroA as a direct AHR ligand. Consistent with other AHR antagonists, UroA failed to induce AHR retention in the nucleus. AHR is necessary for UroA-mediated attenuation of cytokine-induced interleukin 6 (*IL6*) and prostaglandin-endoperoxide synthase 2 (*PTGS2*) expression in Caco-2 cells. Here we identified UroA as the first dietary-derived human selective AHR antagonist produced by the gut microbiota through multi-step metabolism. Furthermore, previously reported anti-inflammatory activity of UroA may at least in part be mediated through AHR.

## 1. Introduction

The aryl hydrocarbon receptor (AHR) is a ligand-activated transcription factor that belongs to basic-helix/loop/helix Per-Arnt-Sim family of proteins. AHR is originally characterized as a xenobiotic receptor that transcriptionally induces drug-metabolizing cytochrome P450 (CYP) enzymes upon activation by xenobiotic compounds, such as 2,3,7,8-tetrachlorodibenzo-*p*-dioxin (TCDD) and polycyclic aromatic hydrocarbons (PAHs) [1]. In the absence of exogenous ligands, AHR resides in the cytoplasm as a complex with heat shock protein 90 (HSP90), X-associated protein 2 (XAP2), and p23. Upon ligand binding, the complex undergoes a conformational transformation, which facilitates translocation to the nucleus, where AHR dimerizes with AHR nuclear translocator (ARNT). AHR:ARNT heterodimer binds to dioxin-response elements (DRE) in the promoter of AHR target genes to modulate gene expression [2]. Recent studies have pointed to a role for the AHR in a myriad of physiological and pathological cellular mechanisms, including development, immunity, epithelial barrier function, and energy homeostasis [3,4]. These findings then led to identification of a number of endogenous and natural AHR ligands, including tryptophan metabolites that exhibit agonist activity, and plant-based flavonoids that are natural antagonists of AHR [5]. 

Ellagitannins (ETs) and ellagic acid (EA) are natural dietary polyphenols found in various fruits and nuts, including walnuts, raspberries, strawberries, pomegranates, and many tropical fruits [6]. There is growing evidence of numerous health benefits of ETs-rich foods. However, ETs are extensively metabolized and the absorption of their hydrolysis product, EA, is also very limited due to its hydrophobic structure [7]. Thus, the beneficial effects of ETs-containing foods are likely attributable to EA-derived metabolites, namely urolithins, that are generated by the gut microbiota [8]. Previous studies have demonstrated micromolar levels of urolithins in the plasma of healthy human subjects and colon tissues of colorectal cancer patients [9] following dietary intake of ETs-rich pomegranate juice and extract [10,11,12,13]. Urolithins have been studied for their anti-oxidant, anti-inflammatory, anti-estrogenic properties and their anti-cancer effects [14,15]. Despite their broad range of health benefits, molecular targets for urolithins remain to be elucidated. Gonzalez-Sarrias et al. showed that Urolithin A (UroA) modulates the expression of numerous genes related to phase I and phase II xenobiotic metabolism, and did not activate *CYP1A1* expression in rat colon, a direct transcriptional target of the AHR [16]. In addition, emerging evidences suggest that AHR could play an important role in tumor growth suppression in colon cancer [17]. Remarkably, Núñez-Sánchez et al. reported that UroA is a major metabolite found in malignant colonic tissues from colorectal cancer patients consuming pomegranate extract. However, these studies did not examine whether UroA interacted with the AHR. In parallel with certain health benefits of urolithins, AHR activation by selective ligands exerts context specific anti-inflammatory and anti-proliferative effects [18,19,20]. Thus, we hypothesized that urolithin metabolites are putative AHR ligands, and it is necessary to investigate whether aforementioned health benefits of urolithins occur in an AHR-dependent manner. 

Here we demonstrated that UroA and UroB exhibit AHR antagonist activity in a species-specific manner, inhibiting human, but not mouse, AHR. We also established that the most abundant urolithin metabolite produced in humans, UroA, is a direct ligand for the AHR as determined using ligand binding competition assays. Furthermore, UroA exerted anti-inflammatory effects that occur through AHR. Thus, our data suggests that UroA is a diet-derived, human-specific AHR ligand, and to our knowledge the first dietary-derived AHR antagonist that is gut-microbiota derived through multi-step metabolism.

## 2. Results

### 2.1. Urolithin Metabolites Do Not Activate AHR-Dependent DRE-Mediated Transcription

**Figure 1:**
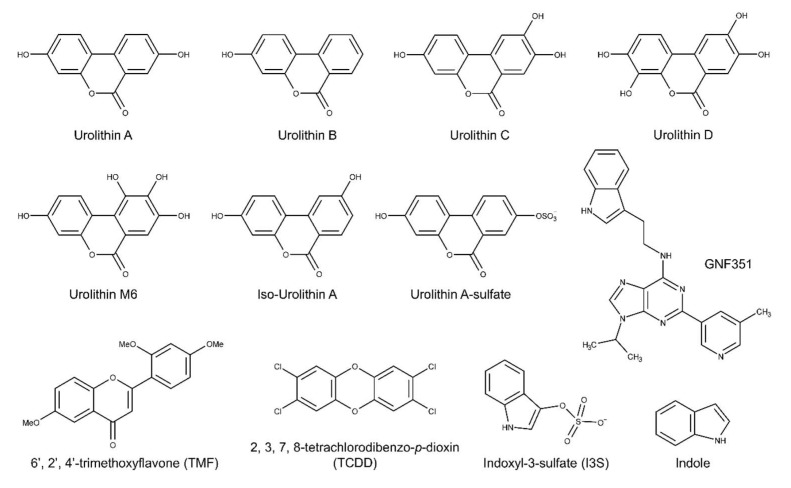
Structures of the urolithins and AHR ligands used in this study.

**Figure 2:**
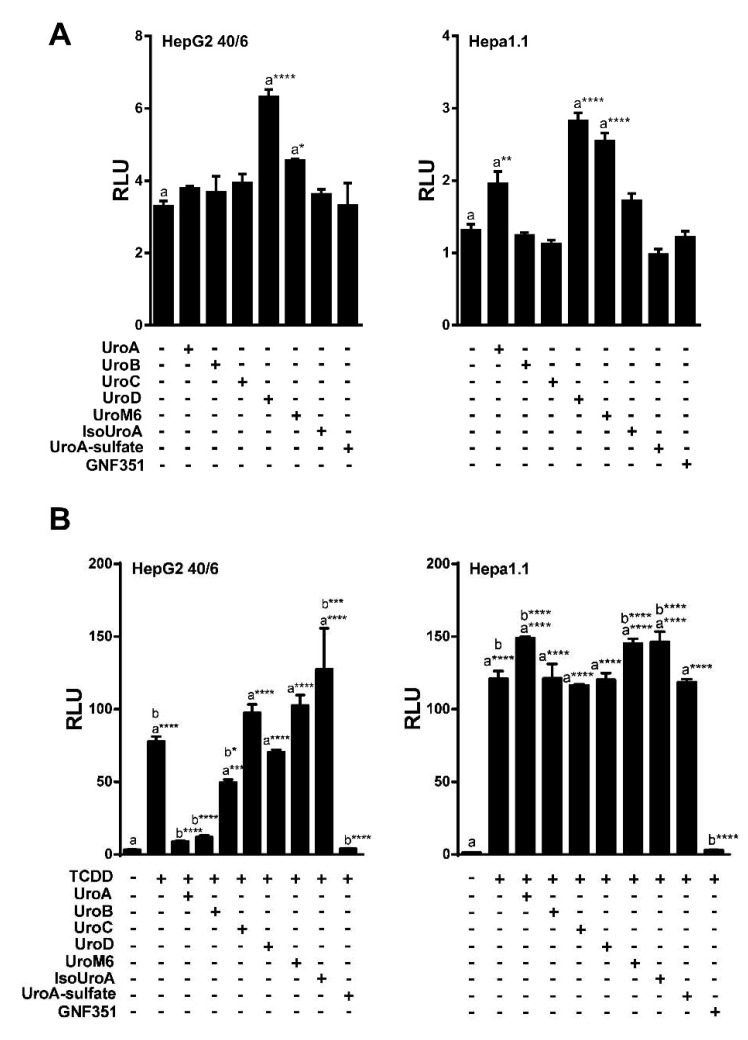
UroA, B and C attenuate TCDD-induced luciferase activity. (**A**) Agonist capacity of urolithin metabolites (10 µM) in human HepG2 (40/6) and mouse Hepa1.1 cell lines. DRE-driven reporter cell lines treated for 4 h with compounds as indicated; (**B**) Antagonist potential of urolithins in reporter cells. HepG2 (40/6) and mouse Hepa1.1 cells were co-treated with TCDD and urolithins (10 µM) for 4 h, lysed, and luciferase activity was measured. Data are presented as mean +/−S.E.M., *n* = 3; significance was determined by one-way ANOVA, *p*-value ≤ 0.01 (**), *p*-value ≤ 0.001 (***) or *p*-value ≤ 0.0001 (****).

To analyze the potential biological activity of urolithin metabolites with regard to AHR-mediated signaling, first we screened urolithin metabolites A, B, C, D, M6, IsoUroA, and A-sulfate for the ability to activate AHR and induce DRE-driven transcriptional activity in cell-based luciferase reporter cell lines. Figure 1 illustrates the structures of urolithin metabolites, as well as established AHR ligands TCDD, GNF351, and TMF. Stable hepatoma-derived human (HepG2 40/6) and mouse (Hepa1.1) cell lines, harboring the pGudluc 6.1 and pGudluc 1.1 DRE-driven luciferase reporter vectors, respectively, were treated with urolithin metabolites as indicated for 4 h, cells were lysed, followed by assessment of luciferase activity. Only urolithins UroD and UroM6 yielded a modest, but statistically significant, increase in luciferase activity, compared with vehicle, in both cell lines at 10 µM (Figure 2A). While 10 µM of UroB exhibited a weak but statistically significant increase in AHR-mediated transcriptional activity in Hepa 1.1 cells. At higher concentrations tested, UroA alone led to a modest increase in luciferase activity in a dose-dependent manner in Hepa1.1 cells (Figure S1a), and to a lesser extent in HepG2 40/6 cells (Figure S2). However, whether it is due to directly activating AHR or blocking the metabolism of endogenous AHR ligands is unclear. 

### 2.2. Urolithins A, B, C Antagonize Ligand-Mediated AHR Transcriptional Activity in a Human-Selective Manner

Next, we tested the ability of urolithin metabolites to antagonize the AHR. The potent AHR agonist TCDD was utilized at a final concentration of 10 nM for HepG2 (40/6) human cells, and 2 nM for Hepa1.1 mouse cells, as the mouse receptor has higher affinity for TCDD relative to human AHR. To determine the antagonist capacity of urolithins, reporter human and mouse cells were co-treated with TCDD and urolithins as indicated for 4 h, followed by cell lysis and luciferase assay. Exposure to TCDD resulted in a >25-fold induction of DRE-dependent luciferase activity compared with vehicle. None of the urolithin metabolites tested exhibited significant antagonism of TCDD-mediated AHR reporter activity in Hepa1.1 cells at 10 µM. In contrast, the AHR antagonist 0.5 µM of GNF351 significantly inhibited TCDD-mediated luciferase activity to control level. In human HepG2 (40/6) cells, 10 µM of UroA, B and C significantly attenuated TCDD-driven AHR activity, UroC being less potent than UroA and B. Reduction of luciferase activity by UroA and UroB was comparable to GNF351-mediated inhibition (Figure 2B). These results demonstrate that UroA, B, and C are potential AHR antagonists. Since UroA and B exhibited the most effective repression of AHR activity, we decided to continue with these two metabolites for further characterization. 

**Figure 3:**
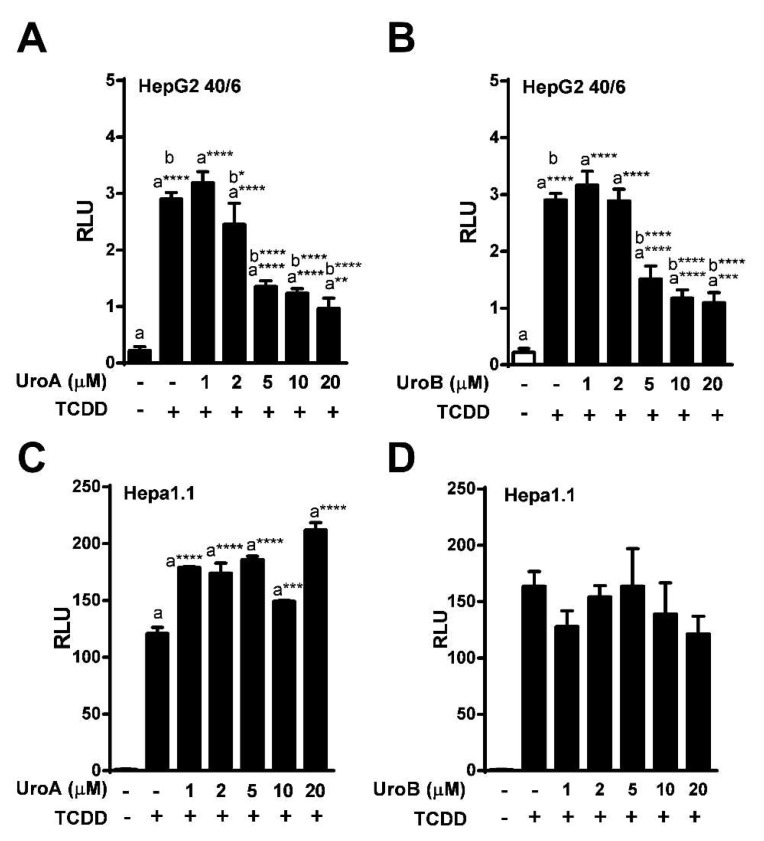
Dose-response assessment of AHR antagonism by UroA and UroB. HepG2 (40/6) reporter cells were treated for 4 h with 10 nM TCDD and indicated doses of (**A**) UroA and (**B**) UroB; (**C** and **D**) Hepa1.1 cells were co-treated with 2 nM TCDD and increasing concentrations of UroA or UroB. Cells were lysed, and luciferase activity was measured. Data are presented as mean +/−S.E.M., *n* = 3; significance was determined by one-way ANOVA, *p*-value ≤ 0.01 (**), *p*-value ≤ 0.001 (***) or *p*-value ≤ 0.0001 (****).

To determine the IC_50_ for UroA and B, HepG2 (40/6) cells were exposed to increasing concentrations of UroA or B (1–20 µM) in combination with TCDD (10 nM) as indicated, for 4 h. AHR-driven luciferase reporter assays demonstrated that exposure to UroA and B led to a dose-dependent attenuation of TCDD-induced relative luciferase activity with an IC_50_ ~ 5 µM (Figure 3A,B). However, UroA failed to antagonize TCDD-mediated AHR activity in mouse Hepa1.1 cells at any dose tested, further indicating species-specific activity of UroA and B on the AHR (Figure 3C, Figure S1b).

**Figure 4:**
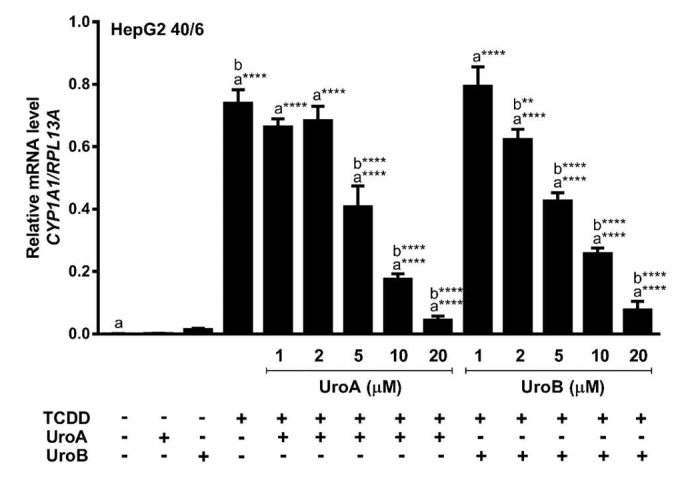
UroA and UroB antagonize AHR-target gene expression. HepG2 (40/6) cells were treated for 4 h with 10 nM TCDD and indicated doses of UroA or UroB. The expression of CYP1A1 was determined by real-time quantitative PCR. Data are presented as mean +/−S.E.M., *n* = 3; significance was determined by one-way ANOVA, *p*-value ≤ 0.0001 (****).

Next, we assessed endogenous AHR target gene expression upon treatment of HepG2 (40/6) cells with increasing concentrations of UroA or B (1–20 µM) in combination with a constant 10 nM TCDD as indicated, for 4 h. Consistent with the reporter-based findings, TCDD alone prompted a robust AHR response with regard to CYP1A1 target gene transcripts (~700-fold increase), whereas combination treatment with UroA or B at 10 µM antagonized TCDD-stimulated CYP1A1 transcription by four-fold and three-fold, respectively. As with the luciferase reporter data, increasing concentrations of UroA or B resulted in a dose-dependent repression of CYP1A1 mRNA levels (Figure 4). The repressive effect of UroA and B was not due to cytotoxicity in HepG2 (40/6) cells (Figure S3a,b).

### 2.3. Endogenous Ligands I3S and Indole Are Antagonized by Urolithin A

**Figure 5:**
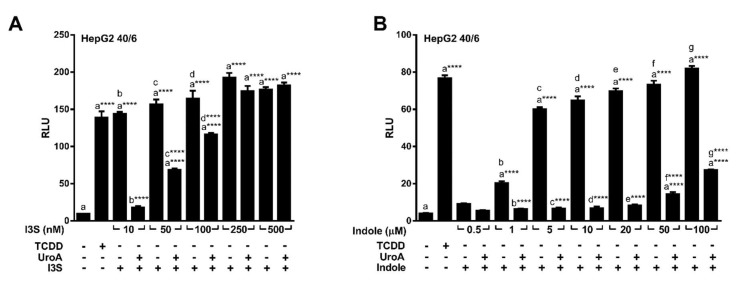
UroA is capable of antagonizing endogenous AHR ligands indole and I3S. HepG2 (40/6) cells were treated for 4 h with 10 µM UroA and indicated doses of (**A**) I3S or (**B**) indole. Cells were lysed, and luciferase activity was measured. Data are presented as mean +/−S.E.M., *n* = 3; significance was determined by one-way ANOVA, *p*-value ≤ 0.0001 (****).

Urolithins are natural dietary-derived metabolites, thus we opted to investigate whether UroA is capable of antagonizing endogenous AHR ligands, indole and indoxyl 3-sulfate (I3S), that exhibit human selectivity. We co-treated HepG2 (40/6) cells with 10 µM UroA and increasing concentrations of I3S or indole for 4 h. Luciferase reporter assays demonstrated that UroA was able to antagonize I3S at up to 100 nM (Figure 5A), and indole even at the highest tested concentration of 100 µM (Figure 5B), which is considerably higher than relevant physiologic indole concentrations [5].

**Figure 6:**
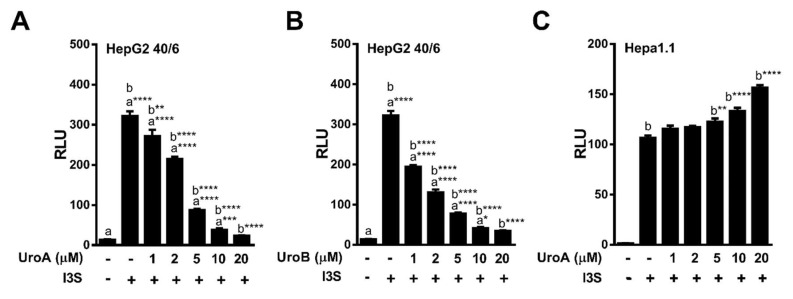
Dose-response antagonism of I3S mediated AHR transcriptional activity by UroA and UroB. (**A**,**B**) HepG2 (40/6) or (**C**) Hepa1.1 reporter cells were treated for 4 h with 10 nM I3S and indicated doses of UroA or UroB. Cells were lysed, and luciferase activity was measured. Data are presented as mean +/−S.E.M., *n* = 3; significance was determined by one-way ANOVA, *p*-value ≤ 0.05 (*), *p*-value ≤ 0.01 (**), *p*-value ≤ 0.001 (***) or *p*-value ≤ 0.001 (***).

Furthermore, increasing concentrations of UroA and B (1 – 20 µM) repressed I3S mediated AHR-driven reporter expression in human HepG2 (40/6) cells in a dose-dependent manner with an IC_50_ ~ 3 µM for UroA, and IC_50_ ~ 2 µM for Uro B (Figure 6A,B). However, exposure of mouse Hepa1.1 cells to UroA failed to recapitulate the attenuation of I3S-induced relative luciferase activity observed with human cells. In contrast, UroA led to a slight increase in reporter expression (Figure 6C). These data indicate that UroA has the capacity to antagonize the prototypic high-affinity ligand TCDD, as well as endogenous human-specific ligands indole and I3S, and exhibits human AHR-selectivity.

### 2.4. Urolithin A Exhibits Sustained Antagonism

**Figure 7:**
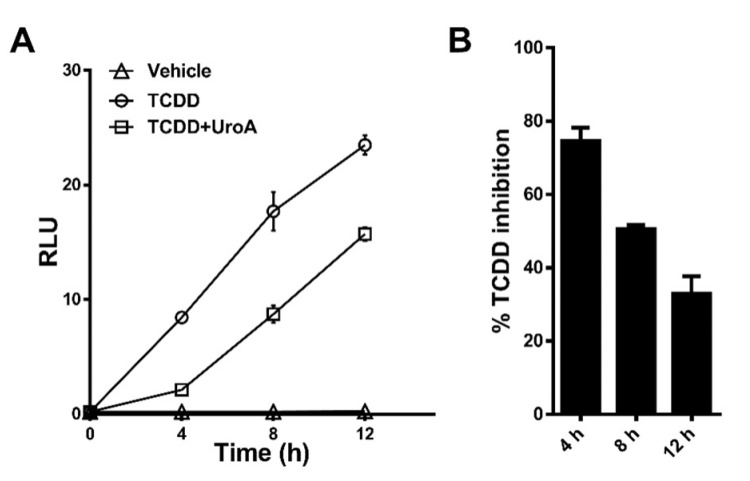
UroA is capable of antagonizing TCDD transcriptional activity up to 12 h. (**A**) HepG2 (40/6) cells were treated for 4, 8, or 12 h with vehicle, 10 nM TCDD and 10 µM UroA as indicated, followed by luciferase reported assay. (**B**) Time-dependent percent inhibition of TCDD-induced luciferase activity by UroA treatment.

To determine the effectiveness of UroA at antagonizing a DRE-driven response over time, we treated HepG2 (40/6) cells with vehicle, TCDD (10 nM) alone or in combination with UroA (10 μM) for 4–8–12 h. TCDD was selected as the AHR agonist because it is essentially not metabolized in mammalian cells. At 4 h, UroA antagonized ~80% of TCDD-induced relative luciferase activity. At 8 h, UroA was able to inhibit ~50% TCDD-mediated AHR activation. By 12 h, UroA still exhibited antagonist activity, but the effectiveness of TCDD inhibition declined to ~30% (Figure 7). These data demonstrate that UroA has the capacity to antagonize AHR with decreasing effectiveness over the course of 12 h.

### 2.5. Urolithin A is a Direct AHR Ligand

**Figure 8:**
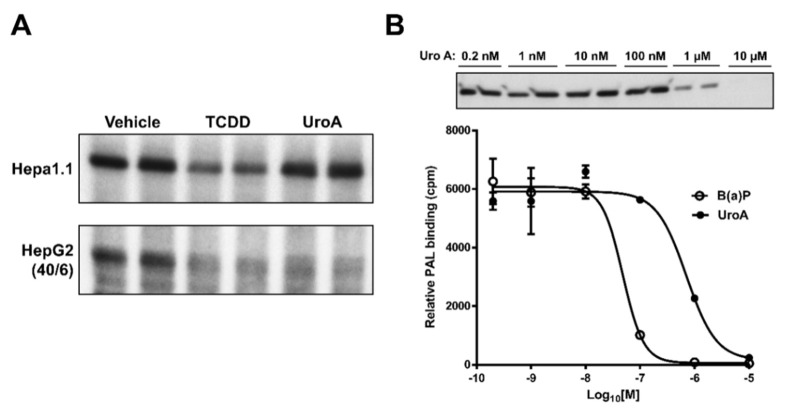
UroA is a direct AHR ligand. Competitive ligand binding assay was performed in (**A**) Hepa1.1 and HepG2 (40/6) cells incubated with the PAL and 10 µM of UroA or TCDD for 1 h, and (**B**) human AHR liver cytosol, in the presence of indicated doses of B(a)P or UroA.

The observations that UroA is capable of inhibiting human AHR activity suggest that UroA is potentially a direct AHR ligand that binds to the ligand binding pocket. To examine whether UroA is a direct AHR ligand, we performed ligand competition binding assays using Hepa1.1 and HepG2 (40/6) cells incubated with photoaffinity ligand (PAL) and 10 µM UroA or the AHR agonist TCDD. UroA was capable of effectively competing with the PAL for binding to human AHR in HepG2 (40/6) cells, at a comparable level as TCDD competition. However, contrary to TCDD, UroA failed to compete with the PAL for binding to mouse AHR in Hepa1.1 cells, indicating human-specific nature of UroA with regard to AHR binding. Next, to determine the IC_50_ for UroA, human AHR-expressing hepatic cytosol was incubated with photoaffinity ligand (PAL) and increasing concentrations of UroA or a known AHR ligand benzo(a)pyrene (B(a)P) for comparison. UroA was capable of effectively competing with the PAL for binding to human AHR in a dose-dependent manner, yielding an IC_50_ = 8.0 × 10^−7^ M. Relative binding affinity of B(a)P for the AHR was IC_50_ = 5.0 × 10^−8^ M, varying by less than one order of magnitude from UroA (Figure 8). These data demonstrate that UroA is a direct ligand of the AHR with a relatively high affinity. 

### 2.6. Urolithin A Does Not Induce AHR Retention in the Nucleus

**Figure 9:**
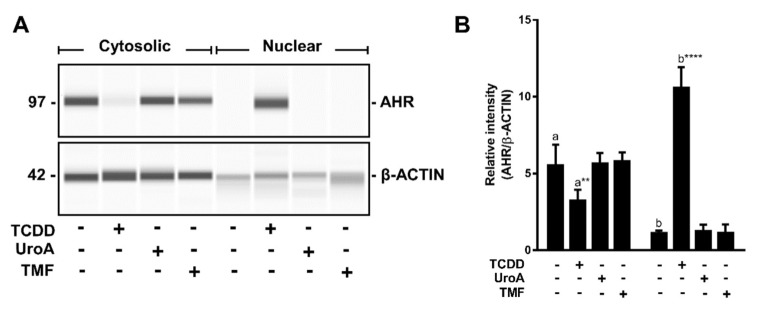
AHR is not retained in the nucleus upon UroA treatment. (**A**) HepG2 (40/6) cells were treated for 1 h with 10 nM TCDD, 10 µM UroA or 10 µM TMF as indicated. AHR protein levels were assessed by Western blot on cytosolic or nuclear extracts; and (**B**) quantification of the immunoblot determined using a ProteinSimple Wes system. Data are presented as mean +/−S.E.M., *n* = 3; significance was determined by one-way ANOVA, *p*-value ≤ 0.01 (**) or *p*-value ≤ 0.0001 (****).

Upon binding to agonists the AHR translocates to the nucleus and forms an AHR/ARNT dimer that binds to DREs in target genes. However, this does not typically occur in the case of antagonist binding. To examine whether UroA binding leads to AHR nuclear retention, we performed immunoblotting assays on sub-cellular fractions isolated from HepG2 (40/6) cells treated with vehicle, TCDD (10 nM), UroA (10 μM) or an established AHR antagonist TMF (10 μM) for 1 h. Cytosolic AHR protein levels were similar in vehicle-, UroA- and TMF-treated samples, and higher than TCDD treatment as agonists prompt nuclear translocation of AHR and subsequent proteolytic turnover. Nuclear AHR levels upon UroA treatment were comparable to that of control and TMF groups, whereas TCDD significantly increased AHR levels in the nucleus. Consistent with other known AHR antagonists, UroA does not cause nuclear retention of the AHR (Figure 9).

### 2.7. Urolithin A Attenuates Cytokine-Induced Inflammatory Signaling in an AHR-Dependent Manner

**Figure 10:**
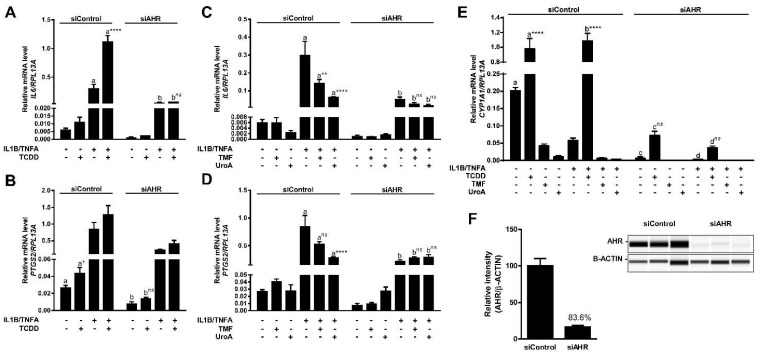
UroA attenuates cytokine-induced inflammatory signaling in an AHR-dependent manner. (**A**,**B**) Caco-2 cells were treated with 10 nM TCDD human cytokines IL1B (20 ng/mL) and TNFA (50 ng/mL) for 4 h; (**C**,**D**) Caco-2 cells were pre-treated with 10 µM TMF or 10 µM UroA for 12 h, followed by exposure to antagonists and human cytokines IL1B (20 ng/mL) and TNFA (50 ng/mL) for 4 h in fresh media. The expressions of IL6 and PTGS2 were assessed by qPCR. Plots in (**A**–**D**) represent different data sets obtained from the same experiment; cells were transfected with 40 nM siControl or siAHR, followed by 48 h incubation before applying treatment; and (**E**) CYP1A1 expression was determined by qPCR in control and AHR-knockdown samples; (**F)** Cells were lysed and AHR expression was assessed in whole cell lysates. Protein levels were quantified by ProteinSimple Wes system, and AHR was normalized to B-ACTIN. Data are presented as mean +/−S.E.M., *n* = 3; significance was determined by one-way ANOVA, *p*-value ≤ 0.01 (**), *p*-value ≤ 0.0001 (****); ns = not significant.

Previous studies reported in vivo anti-inflammatory activity associated with urolithins [21]. We have previously shown that in the presence of inflammatory stimuli AHR antagonists block AHR-mediated enhancement of transcription of specific genes (e.g., *IL6*, *PTGS2*) involved in inflammation [22,23]. Having established UroA as a direct human-selective AHR antagonist, we examined the anti-inflammatory capacity of UroA through inhibition of AHR activity. For this purpose, we utilized the human epithelial colorectal adenocarcinoma cell line Caco-2 as a model cell type since urolithins are produced by the microbiota in the gut. To assess the capacity of UroA to attenuate inflammatory signaling in an AHR-dependent manner, we determined the relative mRNA levels of IL6 and PTGS2. To substantiate previous findings, Caco-2 cells were exposed to TCDD (10 nM) in combination with human cytokines IL1B (20 ng/mL) and TNFA (50 ng/mL) as indicated for 4 h. TCDD exposure alone resulted in a modest induction of IL6 transcription, and cytokines exposure prompted a significantly higher response. As expected, combination treatment with cytokines and TCDD synergistically stimulated IL6 mRNA levels to a greater degree (~5-fold) than with cytokines alone (Figure 10A). Similarly, TCDD and cytokines led to a statistically significant increase of PTGS2 transcript levels, and exposure to a combination of TCDD and cytokines resulted in a slightly higher PTGS2 induction, albeit not statistically significant (Figure 10B). Next, Caco-2 cells were stimulated for 4 h with human cytokines IL1B (20 ng/mL) and TNFA (50 ng/mL) following a 12 h pre-incubation with vehicle, UroA (10 μM) or the AHR antagonist TMF (10 μM), as indicated. UroA was capable of attenuating both basal and cytokine-induced mRNA levels of IL6, while TMF was able to repress only cytokine-induced IL6, but not basal IL6 levels (Figure 10C). Similarly, UroA significantly inhibited cytokine-induced PTGS2 more effectively than TMF (Figure 10D). UroA-mediated repression of inflammatory mediators was not due to cytotoxicity in Caco-2 cells (Figure S3C).

To examine whether anti-inflammatory function of UroA is mediated through AHR, we utilized siRNA to repress AHR expression in Caco-2 cells. Western blot analysis demonstrated an >80% decrease in AHR protein expression (Figure 10F). Consistent with this, we were able to observe a functional effect of decreased AHR levels with regard to CYP1A1 transcription. Basal CYP1A1 mRNA levels were diminished ~30-fold in siAHR-transfected cells compared with siControl-transfected cells. There was a ~13-fold decrease in TCDD-induced CYP1A1 in cells exhibiting lower AHR expression relative to control cells (Figure 10E). Attenuation of AHR expression also resulted in a loss of inflammatory gene expression mediated by UroA and TMF, as well as, the transcriptional stimulatory effect of TCDD on both IL6 and PTGS2 expression, indicating that UroA exerts its anti-inflammatory function in an AHR-dependent manner. Interestingly, basal expression and cytokine-induced levels of IL6 and PTGS2 were also diminished in siAHR-transfected cells (Figure 10A–D).

## 3. Discussion

Recent studies have pointed to a role of AHR in a myriad of cellular mechanisms, including cell cycle, tumor invasiveness and immune function. Discovery of its numerous physiological functions raised the hypothesis of the existence of endogenous ligands for AHR. Over the last decade, a number of endogenous and natural AHR ligands has been identified, including the agonists; microbiota-derived indoles [24], tryptophan metabolites kynurenic acid [25], indoxyl sulfate [26], indirubin [27], as well as plant-derived flavonoids, many of which are dietary AHR antagonists [28,29]. Despite the variety of endogenous AHR agonists, identification of natural AHR antagonists is limited. Dietary flavonoids have been studied for their capacity to exert AHR activity. Various structurally diverse phytochemicals displayed AHR agonist or antagonist activity, depending on their structure and the cell context [28]. In this study, we demonstrate that UroA is the first identified dietary-derived human-specific AHR antagonist produced by gut microbiota. 

Both similarities and differences can be found in the metabolism of urolithins and flavonoids in humans to generate an AHR ligands. Flavonoids, upon ingestion, undergo deglycosylation via β-glucosidase activity in the intestinal tract and are absorbed [30,31]. However, absorption of certain flavonoids that are coupled with rhamnosyl moieties require their hydrolysis in the colon by α-rhamnosidases secreted by *Bifidobacterium dentium*, a member of the human microflora [32]. In contrast, urolithins are produced from the catabolism of the dietary polymeric polyphenols ellagitannins, and ellagic acid catalyzed by the gut microbiota through a multi-step metabolism rather than a deconjugation reaction. Previous studies reported the involvement of gut microbiota in the production of urolithins by revealing that germ-free rats orally administrated with ellagic acid failed to synthesize urolithins [33]. Furthermore, production of urolithins by human gut microbiota was demonstrated for the first time by incubation of human fecal samples with ellagic acid, the ellagitannin punicalagin and an ellagitannin-rich walnut extract under anaerobic conditions [13]. Studies on the Iberian pig examining urolithin production in the gut elucidated that one of the lactone rings of ellagic acid is first removed to produce tetrahydroxy-urolithin, followed by removal of hydroxyls to yield UroA and UroB [34]. Urolithins are readily absorbed and can circulate in human plasma at relatively high concentrations. Indeed, urolithins were detected in plasma of healthy humans 6 – 8 h after intake of ellagitannin-rich pomegranate juice, suggesting that production occurs in the colon. The peak plasma levels of UroA in these volunteers were 14–25 μM [10,11,12,13]. In addition, high micromolar concentrations of urolithins have been found in the colon lumen of colorectal cancer patients [9] and feces of healthy suggests [35] consuming ETs-rich pomegranate extract. Thus, it is noteworthy that urolithin doses utilized in our study are within the physiologically relevant concentration range.

Numerous studies point to anti-inflammatory activities associated with urolithins [21]. Oral administration of UroA attenuated inflammatory signaling and preserved colonic architecture in a DSS-induced rat colitis model [36], and diminished carrageenan-induced paw edema in mice [37]. A number of inflammatory mediators, including cytokines *IL6*, *IL1B*, chemokines *Cxcl5*, *Ccl20*, and prostaglandin-endoperoxide synthase *PTGS2* are transcriptionally regulated by AHR [25,38,39,40]. Antagonism or selective activation of AHR has been demonstrated to suppress TPA-induced ear edema in mice, repress inflammatory signaling in primary human fibroblast-like synoviocytes, murine peritoneal macrophages, human head and neck tumor cells [18,19,22,23]. Thus, we opted to investigate whether anti-inflammatory activity of urolithins is mediated in part through an AHR-dependent mechanism. We demonstrate that UroA inhibits both basal and cytokine-induced *IL6* and *PTGS2* mRNA levels in human Caco-2 cell line. AHR knockdown by siRNA prevented the UroA repression of inflammatory mediators, suggesting that anti-inflammatory characteristic of UroA is mediated, at least in part, through AHR. However, it is important to note that multiple pathways have been reported to contribute to the anti-inflammatory activities of urolithins, including, c-Jun [41], NF-KB/AP1 [42] and MAPK [43]. The role of urolithins as potential in vivo antioxidants is controversial [10]. Nevertheless, we cannot completely rule out the contribution of antioxidant effect on the inhibition of inflammatory signaling by urolithins. Indeed, urolithins have been shown to exhibit antioxidant activity in Caco-2 cells, although at higher concentrations than are required to inhibit AHR in our studies [44]. Thus, it is likely that AHR is one of the multiple receptors involved in mediating the effects of UroA. Furthermore, cytokine treatment led to an AHR-dependent upregulation of *IL-6* and *COX-2*. This might be explained by a cytokine-mediated decrease in *CYP1A1* levels, leading to an accumulation of CYP1A1 substrates (i.e., AHR ligands). This, in turn, may lead to AHR-dependent upregulation of *IL-6* and *COX-2*.

Genetic ablation of AHR expression results in certain developmental defects in vasculature, immune and reproductive systems as observed with AHR-deficient mice [45,46,47,48]; however, transgenic mice expressing a constitutively activated AHR also have a number of complications, including invasive stomach tumors, thymus atrophy, liver enlargement, and increased mortality [49]. Furthermore, a wide variety of tumor types exhibit enhanced AHR expression with constitutive occurrence in the nucleus, suggesting a persistently activated AHR in tumors [50]. We are constantly being exposed to AHR agonists that are found in our environment, diet, and produced by gut microbiota. This becomes particularly more critical in a tumor environment. For instance, colonic tumor cells are locally exposed to gut microbiota-derived indole [24], indole-3-aldehyde [51], and IDO pathway-generated tryptophan metabolites and kynurenic acid [25]. Additionally, systemically circulating endogenous ligands, including indoxyl sulfate [26] and indirubin [27], as well as exposure to potent environmental ligands in combination with increased AHR expression in tumor cells, result in constant activation of AHR. Consequently, persistent AHR activation may lead to tumor progression and escape from tumor immune surveillance through stimulation of regulatory T cell production in the tumor environment [50]. Human glioma cells have been demonstrated to release high levels of kynurenine by constitutively degrading tryptophan through the tryptophan-2,3-dioxygenase (TDO) pathway. Furthermore, elevated TDO expression positively correlated with proliferation index and malignancy in human brain tumor samples. Kynurenine production by glioma-derived TDO inhibited anti-tumor immune responses and augmented survival and motility of tumor cells in an AHR-dependent manner [52]. Similarly, triple negative (ER-/PR-/Her2-) human breast cancer cells exhibit elevated TDO2 expression that is dependent on the AHR. Consequently, excess intracellular kynurenine produced by this amplification loop promotes tumor cell migration [53]. Consistent with this, AHR antagonist CB7993113 suppressed tumor growth and increased overall survival in vivo in an oral squamous cell carcinoma mouse model [54]. Furthermore, our group previously demonstrated that AHR antagonism attenuates aggressive phenotypes in head and neck tumor cell lines [55,56]. These studies point towards the risks of uninterrupted excessive AHR activation, and in particular in combination with inflammatory signaling such as Toll-like receptor activation or cytokine release in the tumor microenvironment. Thus, it is critical to have balanced AHR activity for overall homeostasis and prevention of certain pathological conditions that are known to correlate with elevated AHR activity such as cancer. Antagonizing AHR with natural sources, such as dietary consumption of ellagitannin-enriched foods, therefore, will be key to limit overactivation of the AHR. It is noteworthy that certain endogenous and gut microbiota-derived AHR agonists favor human AHR activation and is conserved in primates [57,58]. Recently, Faber, et al. showed increased potency of endogenous AHR ligand indirubin upon mutating H326 and A349 residues in the mouse AHR ligand-binding domain to the corresponding residues in the human AHR ligand-binding domain. These mutations did not affect ligand binding; instead likely enhanced the efficiency of transformation/DNA binding [59]. Thus, the species-specific responses observed in this report could be attributed to binding affinity or transformation efficiency of the receptor upon ligand binding. Similarly, UroA exhibits human AHR selectivity, and is capable of antagonizing indole and I3S at physiologically relevant concentrations. Moreover, in the absence of exogenous ligands, UroA does not activate basal transcription of *CYP1A1* as well as DRE-driven luciferase expression, indicating its capacity to antagonize endogenous AHR activity. Thus, formation of urolithin metabolites, particularly UroA, is of great importance in disease states in view of human relevancy. 

Urolithins have been studied for their anti-cancer properties. In a wide variety of human cancer lines, including colon, kidney, prostate, liver, breast, bladder, and lymphatic cancer, urolithins were capable of inhibiting cell proliferation, blocking cell cycle, and inducing apoptosis. Key modulators of cell cycle and proliferation such as p53, CMYC, cyclins, as well as signaling pathways critical in tumorigenesis, including Wnt/β-catenin and EGFR signaling, were influenced by urolithins [60,61]. Tumor suppressor p21 was upregulated [62], and phase I drug metabolizing enzymes were repressed by urolithins in colorectal cancer cells [63], which might be preventive against biotransformation of polycyclic aromatic hydrocarbons in the colon. Thus, it is plausible that there is a close link between the anti-cancer effects of urolithins and AHR antagonism in humans. However, other mechanisms for urolithin-mediated anti-cancer effects have been proposed [64], such as PI3K/AKT/mTOR [65] and AR receptor [66]. Further in vivo studies are certainly required for assessing the ability of urolithins to control human tumor growth, and the role of AHR in mediating these effects.

In summary, we report here for the first time the identification and characterization of UroA as a natural microbiota-derived human-selective AHR ligand. UroA has the capacity to effectively repress AHR-mediated transcription through antagonism of endogenous and exogenous AHR agonists. Moreover, previously reported anti-inflammatory characteristic of UroA can be at least partly attributable to AHR. It is noteworthy that UroA reveals species dependency with regard to functional AHR antagonism and may have applications both therapeutically and for the further investigation of AHR function.

## 4. Materials and Methods

### 4.1. Chemicals and Reagents

2,3,7,8-tetrachlorodibenzo-*p*-dioxin (TCDD) was kindly provided by Dr. Stephen Safe (Texas A and M University, College Station, TX, USA). Urolithins were synthesized as previously described [67]. 6,2’,4’-trimethoxyflavone (TMF) was purchased from INDOFINE Chemical Company, Inc. (Hillsborough, NJ, USA). Indole was purchased and re-purified as previously described [24]. Recombinant human tumor necrosis factor alpha (TNFA) and human IL1B were purchased from PeproTech (Rocky Hill, NJ, USA). 

### 4.2. Cell Culture

HepG2 (40/6) cells were generated as previously described [68]. Hepa1.1 cells were a gift from Dr. Michael Denison (University of California, Davis, CA, USA) and Caco-2 cells were obtained from American Type Culture Collection. The stable reporter cell lines human HepG2 (40/6) and mouse Hepa1.1, as well as the human epithelial colorectal adenocarcinoma cell line Caco-2, were maintained in α-minimal essential medium (Sigma, St. Louis, MO, USA), supplemented with 10% fetal bovine serum (HyClone Laboratories, Logan, UT, USA), 100 U/mL penicillin, and 100 μg/mL streptomycin (Sigma, St. Louis, MO, USA). Cells were grown in a humidified incubator at 37 °C, with an atmospheric composition of 95% air and 5% CO_2_.

### 4.3. Luciferase Reporter Assays

The AHR reporter cell lines used in luciferase reporter assays were seeded in twelve-well plates and treated the following day with AHR ligands (TCDD, indole, 3-indoxyl-sulfate I3S, N-(2-(1H-indol-3-yl)ethyl)-9-isopropyl-2-(5-methylpyridin-3-yl)-9H-purin-6-amine GNF351), and urolithin metabolites dissolved in dimethyl sulfoxide (DMSO) (0.1% final concentration in cell culture) and incubated for 4 h. Cells were then lysed with lysis buffer [25 mM Tris-phosphate, pH 7.8, 2 mM dithiothreitol, 2 mM 1,2-diaminhocyclohexane-N,N,N′,N′-tetraacetic acid, 10% (*v*/*v*) glycerol, and 1% (*v*/*v*) Triton X-100]. The luciferase activity was measured in the lysate after freeze-thaw using a TD-20e luminometer and luciferase assay substrate (Promega, Madison, WI, USA) according to the manufacturer’s instructions.

### 4.4. PAL Ligand Competition Assay

The AHR photoaffinity ligand (PAL), 2-azido-3-[^125^I]iodo-7,8-dibromodibenzo-*p*-dioxin, was synthesized and competition binding assays performed essentially as described previously [57]. Briefly, PAL was added directly to cultured cells, and incubated for 1 h with 10 µM of UroA or 10 nM of TCDD for HepG2 (40/6) cells, or 2 nM of TCDD for Hepa1.1 cells. The cells were then UV photolyzed (402 nm), lysed, and lysates were subjected to gel electrophoresis on an 8% tricine-polyacrylamide gel, followed by transfer to a polyvinylidene difluoride membrane and visualization by autoradiography.

For IC_50_ assessment, PAL was added to cytosolic extracts obtained from mouse liver that expresses the human AHR, and incubated with increasing concentrations of UroA or benzo(a)pyrene at room temperature for 30 min. The samples were then UV photolyzed (402 nm), and extracts were incubated with 1% charcoal/dextran (final concentration) and centrifuged to remove remaining unbound PAL. The samples were subjected to gel electrophoresis on an 8% tricine-polyacrylamide gel, transferred to a polyvinylidene difluoride membrane and visualized by autoradiography. Radioactive bands were cut from the membrane for gamma isotope quantification.

### 4.5. RNA Isolation and Quantitative Real-Time PCR Analysis

Total RNA was isolated using TRI Reagent (Sigma-Aldrich, St. Louis, MO), and cDNA was synthesized using a high capacity cDNA reverse transcription kit (Applied Biosystems). Quantitative real-time PCR was performed using PerfeCTa SYBR Green Supermix for iQ (Quanta Biosciences, Beverly, MA, USA) on a CFX Connect Real-Time PCR Detection System (Bio-Rad, Hercules, CA, USA). Primers used for real-time PCR are listed in Table 1.

### 4.6. AHR Nuclear Translocation Analysis

Cytosolic and nuclear extracts were prepared as previously described [18]. Briefly, after 1 h ligand treatment, cells were washed and scraped into PBS. Cell pellets were resuspended in MENG (25 mM MOPS, 2 mM EDTA, 0.02% sodium azide, and 10% glycerol, pH 7.4) containing protease inhibitor cocktail (Roche) and homogenized with a stainless-steel Dura-Grind Dounce homogenizer (Wheaton Instruments, Millville, NJ, USA). Cell homogenates were centrifuged at 1000× *g* for 20 min, and the supernatant was then subjected to ultracentrifugation to generate cytosol. The nuclear pellet was washed three times with homogenization buffer, and then extracted with MENG containing 500 mM NaCl for 1 h, followed by centrifugation. Cell extracts were resolved on 8% tricine sodium dodecyl sulfate-polyacrylamide gel electrophoresis, and proteins were transferred to PVDF membrane. Specific proteins were detected using anti-AHR (Thermo Fisher Scientific, Inc., Waltham, MA, USA), or anti-actin (Santa Cruz Biotechnology, Santa Cruz, CA, USA) primary antibodies, and visualized with species appropriate biotin-conjugated secondary antibodies (Jackson Immunoresearch, West Grove, PA, USA) and a subsequent incubation with ^125^I-streptavidin, which was generated as described [69].

### 4.7. Gene Silencing

Caco-2 cells were transfected with siAHR or siControl (Dharmacon, Lafayette, CO, USA) using Lipofectamine 3000 Transfection Reagent (Thermo Fisher Scientific, Inc., Waltham, MA, USA) according to manufacturer’s instructions. 48 h after transfection, Caco-2 cells were pre-treated with UroA (10 µM) or TMF (10 µM) for 12 h, and then media was changed, cells were re-treated with UroA or TMF, as well as TCDD (10 nM) and human cytokines IL1B (10 ng/mL) and TNFA (50 ng/mL) for 4 h. 

### 4.8. Statistical Analysis

Data were analyzed using GraphPad Prism 6 software, (GraphPad Software, Inc., San Diego, CA, USA). One-way or two-way ANOVA analysis was used with Tukey’s multiple comparison post test to determine statistical significance between treatments. Data represent mean ± S.E.M. and are representative of three independent experiments *p*-value ≤ 0.05 (*), *p*-value ≤ 0.01 (**), *p*-value ≤ 0.001 (***) and *p*-value ≤ 0.0001 (****).

## Figures and Tables

**Table 1:** Primers for real-time PCR.

Primer Name	Primer Sequence
CYP1A1	5′-TACCTCAGCCACCTCCAAGAT-3′ 5′-GAGGTCTTGAGGCCCTGAT-3′
IL6	5′-AAATTCGGTACATCCTCGACG-3′ 5′-AGTGCCTCTTTGCTGCTTTCA-3′
PTGS2	5′-CAAATCCTTGCTGTTCCCACCCAT-3′ 5′-GTGCACTGTGTTTGGAGTGGGTTT-3′
RPL13A	5′-CCTGGAGGAGAAGAGGAAAGAGA-3′ 5′-GAGGACCTCTGTGTATTTGTCA-3′

## Supplementary Materials

The following are available online at http://www.mdpi.com/2218-1989/8/4/86/s1, Figure S1: The agonist and antagonist activity of UroA was assessed in Hepa1.1 at higher doses, Figure S2: The agonist activity of UroA was assessed in HepG2 40/6 at higher doses, Figure S3: UroA does not cause cytotoxicity in Hepa1.1, HepG2 (40/6) or Caco-2 cells.

## References

[1] Durrin L.K., Jones P.B.C., Fisher J.M., Galeazzi D.R., Whitlock J.P. (1987). 2,3,7,8-Tetrachlorodibenzo-p-dioxin receptors regulate transcription of the cytochrome P1-450 gene. J. Cell. Biochem..

[2] Rowlands J.C., Gustafsson J.Å. (1997). Aryl hydrocarbon receptor-mediated signal transduction. Crit. Rev. Toxicol..

[3] Hao N., Whitelaw M.L. (2013). The emerging roles of AhR in physiology and immunity. Biochem. Pharmacol..

[4] Murray I.A., Perdew G.H. (2016). Ligand activation of the Ah receptor contributes to gastrointestinal homeostasis. Curr. Opin. Toxicol..

[5] Hubbard T.D., Murray I.A., Perdew G.H. (2015). Indole and tryptophan metabolism: Endogenous and dietary routes to Ah receptor activation. Drug Metab. Dispos..

[6] Espín J.C., Larrosa M., García-Conesa M., Tomás-Barberán F. (2013). Biological significance of the gut microbial ellagic acid-derived metabolites urolithins. Evid.-Based Complement. Altern. Med..

[7] González-Sarrías A., García-Villalba R., Núñez-Sánchez M.Á., Tomé-Carneiro J., Zafrilla P., Mulero J., Tomás-Barberán F.A., Espín J.C. (2015). Identifying the limits for ellagic acid bioavailability: A crossover pharmacokinetic study in healthy volunteers after consumption of pomegranate extracts. J. Funct. Foods.

[8] Cerdá B., Tomás-Barberán F.A., Espín J.C. (2005). Metabolism of antioxidant and chemopreventive ellagitannins from strawberries, raspberries, walnuts, and oak-aged wine in humans: Identification of biomarkers and individual variability. J. Agric. Food Chem..

[9] Nuñez-Sánchez M.A., García-Villalba R., Monedero-Saiz T., García-Talavera N.V., Gómez-Sánchez M.B., Sánchez-Álvarez C., García-Albert A.M., Rodríguez-Gil F.J., Ruiz-Marín M., Pastor-Quirante F.A. (2014). Targeted metabolic profiling of pomegranate polyphenols and urolithins in plasma, urine and colon tissues from colorectal cancer patients. Mol. Nutr. Food Res..

[10] Cerdá B., Espín J.C., Parra S., Martínez P., Tomás-Barberán F.A. (2004). The potent in vitro antioxidant ellagitannins from pomegranate juice are metabolised into bioavailable but poor antioxidant hydroxy-6H-dibenzopyran-6-one derivatives by the colonic microflora of healthy humans. Eur. J. Nutr..

[11] Seeram N.P., Henning S.M., Zhang Y., Suchard M., Li Z., Heber D. (2006). Pomegranate juice ellagitannin metabolites are present in human plasma and some persist in urine for up to 48 hours. J. Nutr..

[12] Tomás-Barberan F.A., Espín J.C., García-Conesa M.T. (2009). Bioavailability and metabolism of ellagic acid and ellagitannins. Chemistry and Biology of Ellagitannins.

[13] Cerdá B., Periago P., Espín J.C., Tomás-Barberán F.A. (2005). Identification of Urolithin A as a metabolite produced by human colon microflora from ellagic acid and related compounds. J. Agric. Food Chem..

[14] Larrosa M., García-Conesa M.T., Espín J.C., Tomás-Barberán F.A. (2010). Ellagitannins, ellagic acid and vascular health. Mol. Asp. Med..

[15] Heber D. (2008). Multitargeted therapy of cancer by ellagitannins. Cancer Lett..

[16] González-Sarrías A., Azorín-Ortuño M., Yáñez-Gascón M.J., Tomás-Barberán F.A., García-Conesa M.T., Espín J.C. (2009). Dissimilar in vitro and in vivo effects of ellagic acid and its microbiota-derived metabolites, urolithins, on the cytochrome P450 1A1. J. Agric. Food Chem..

[17] Ikuta T., Kurosumi M., Yatsuoka T., Nishimura Y. (2016). Tissue distribution of aryl hydrocarbon receptor in the intestine: Implication of putative roles in tumor suppression. Exp. Cell Res..

[18] Muku G.E., Lahoti T.S., Murray I.A., Podolsky M.A., Smith K.J., Hubbard T.D., Kuzu G., Gowda K., Amin S.G., Perdew G.H. (2017). Ligand-mediated cytoplasmic retention of the Ah receptor inhibits macrophage-mediated acute inflammatory responses. Lab. Investig..

[19] Murray I.A., Krishnegowda G., DiNatale B.C., Flaveny C., Chiaro C., Lin J.M., Sharma A.K., Amin S., Perdew G.H. (2010). Development of a selective modulator of aryl hydrocarbon (Ah) receptor activity that exhibits anti-inflammatory properties. Chem. Res. Toxicol..

[20] Dietrich C. (2011). The AHR in the control of cell cycle and apoptosis. The Ah Receptor in Biology and Toxicology.

[21] Tomás-Barberán F.A., González-Sarrías A., García-Villalba R., Núñez-Sánchez M.A., Selma M.V., García-Conesa M.T., Espín J.C. (2017). Urolithins, the rescue of “old” metabolites to understand a “new” concept: Metabotypes as a nexus among phenolic metabolism, microbiota dysbiosis, and host health status. Mol. Nutr. Food Res..

[22] DiNatale B.C., Schroeder J.C., Perdew G.H. (2011). Ah receptor antagonism inhibits constitutive and cytokine inducible IL6 production in head and neck tumor cell lines. Mol. Carcinog..

[23] Lahoti T.S., John K., Hughes J.M., Kusnadi A., Murray I.A., Krishnegowda G., Amin S., Perdew G.H. (2013). Aryl hydrocarbon receptor antagonism mitigates cytokine-mediated inflammatory signalling in primary human fibroblast-like synoviocytes. Ann. Rheum. Dis..

[24] Hubbard T.D., Murray I.A., Bisson W.H., Lahoti T.S., Gowda K., Amin S.G., Patterson A.D., Perdew G.H. (2015). Adaptation of the human aryl hydrocarbon receptor to sense microbiota-derived indoles. Sci. Rep..

[25] DiNatale B.C., Murray I.A., Schroeder J.C., Flaveny C.A., Lahoti T.S., Laurenzana E.M., Omiecinski C.J., Perdew G.H. (2010). Kynurenic acid is a potent endogenous aryl hydrocarbon receptor ligand that synergistically induces interleukin-6 in the presence of inflammatory signaling. Toxicol. Sci..

[26] Schroeder J.C., DiNatale B.C., Murray I.A., Flaveny C.A., Liu Q., Laurenzana E.M., Lin J.M., Strom S.C., Omiecinski C.J., Amin S. (2010). The uremic toxin 3-indoxyl sulfate is a potent endogenous agonist for the human aryl hydrocarbon receptor. Biochemistry.

[27] Adachi J., Mori Y., Matsui S., Takigami H., Fujino J., Kitagawa H., Miller C.A., Kato T., Saeki K., Matsuda T. (2001). Indirubin and indigo are potent aryl hydrocarbon receptor ligands present in human urine. J. Biol. Chem..

[28] Zhang S., Qin C., Safe S.H. (2003). Flavonoids as aryl hydrocarbon receptor agonists/antagonists: Effects of structure and cell context. Environ. Health Perspect..

[29] Murray I.A., Flaveny C.A., DiNatale B.C., Chairo C.R., Schroeder J.C., Kusnadi A., Perdew G.H. (2010). Antagonism of aryl hydrocarbon receptor signaling by 6,2’,4’-trimethoxyflavone. J. Pharmacol. Exp. Ther..

[30] Day A.J., Cañada F.J., Díaz J.C., Kroon P.A., Mclauchlan R., Faulds C.B., Plumb G.W., Morgan M.R., Williamson G. (2010). Dietary flavonoid and isoflavone glycosides are hydrolysed by the lactase site of lactase phlorizin hydrolase. FEBS Lett..

[31] Day A.J., DuPont M.S., Ridley S., Rhodes M., Rhodes M.J., Morgan M.R., Williamson G. (1998). Deglycosylation of flavonoid and isoflavonoid glycosides by human small intestine and liver β-glucosidase activity. FEBS Lett..

[32] Bang S.H. (2015). Metabolism of rutin and poncirin by human intestinal microbiota and cloning of their metabolizing α-L-rhamnosidase from Bifidobacterium dentium. J. Microbiol. Biotechnol..

[33] Doyle B., Griffiths L.A. (1980). The metabolism of ellagic acid in the rat. Xenobiotica.

[34] Espín J.C., González-Barrio R., Cerdá B., López-Bote C., Rey A.I., Tomás-Barberán F.A. (2007). Iberian pig as a model to clarify obscure points in the bioavailability and metabolism of ellagitannins in humans. J. Agric. Food Chem..

[35] González-Sarrías A., García-Villalba R., Romo-Vaquero M., Alasalvar C., Örem A., Zafrilla P., Tomás-Barberán F.A., Selma M.V., Espín J.C. (2017). Clustering according to urolithin metabotype explains the interindividual variability in the improvement of cardiovascular risk biomarkers in overweight-obese individuals consuming pomegranate: A randomized clinical trial. Mol. Nutr. Food Res..

[36] Larrosa M., González-Sarrías A., Yáñez-Gascón M.J., Selma M.V., Azorín-Ortuño M., Toti S., Tomás-Barberán F., Dolara P., Espín J.C. (2010). Anti-inflammatory properties of a pomegranate extract and its metabolite urolithin-A in a colitis rat model and the effect of colon inflammation on phenolic metabolism. J. Nutr. Biochem..

[37] Ishimoto H., Shibata M., Myojin Y., Ito H., Sugimoto Y., Tai A., Hatano T. (2011). In vivo anti-inflammatory and antioxidant properties of ellagitannin metabolite urolithin A. Bioorg. Med. Chem. Lett..

[38] Smith K.J., Boyer J.A., Muku G.E., Murray I.A., Gowda K., Desai D., Amin S.G., Glick A.B., Perdew G.H. (2017). Editor’s Highlight: Ah receptor activation potentiates neutrophil chemoattractant (C-X-C Motif) ligand 5 expression in keratinocytes and skin. Toxicol. Sci..

[39] Lahoti T.S., Boyer J.A., Kusnadi A., Muku G.E., Murray I.A., Perdew G.H. (2015). Aryl hydrocarbon receptor activation synergistically induces lipopolysaccharide-mediated expression of proinflammatory chemokine (c–c motif) ligand 20. Toxicol. Sci..

[40] Yang F., Bleich D. (2004). Transcriptional regulation of cyclooxygenase-2 gene in pancreatic β-cells. J. Biol. Chem..

[41] Liu H., Kang H., Song C., Lei Z., Li L., Guo J., Xu Y., Guan H., Fang Z., Li F. (2018). Urolithin A Inhibits the Catabolic Effect of TNFα on Nucleus Pulposus Cell and Alleviates Intervertebral Disc Degeneration in vivo. Front. Pharmacol..

[42] Komatsu W., Kishi H., Yagasaki K., Ohhira S. (2018). Urolithin A attenuates pro-inflammatory mediator production by suppressing PI3-K/Akt/NF-κB and JNK/AP-1 signaling pathways in lipopolysaccharide-stimulated RAW264 macrophages: Possible involvement of NADPH oxidase-derived reactive oxygen species. Eur. J. Pharmacol..

[43] Xu J., Yuan C., Wang G., Luo J., Ma H., Xu L., Mu Y., Li Y., Seeram N.P., Huang X. (2018). Urolithins Attenuate LPS-Induced Neuroinflammation in BV2 Microglia via MAPK, Akt, and NF-κB Signaling Pathways. J. Agric. Food Chem..

[44] Kojadinovic M., Arsic A., Petovic-Oggiano G., Gavrovic-Jankulovic M., Glibetic M., Popovic M. (2017). Effect of urolithins on oxidative stress of colorectal adenocarcinomacells-Caco-2. Int. J. Food Sci. Nutr..

[45] Lahvis G.P., Lindell S.L., Thomas R.S., McCuskey R.S., Murphy C., Glover E., Bentz M., Southard J., Bradfield C.A. (2000). Portosystemic shunting and persistent fetal vascular structures in aryl hydrocarbon receptor-deficient mice. Proc. Natl. Acad. Sci. USA.

[46] Lahvis G.P., Pyzalski R.W., Glover E., Pitot H.C., McElwee M.K., Bradfield C.A. (2004). The aryl hydrocarbon receptor is required for developmental closure of the ductus venosus in the neonatal mouse. Mol. Pharmacol..

[47] Fernandez-Salguero P., Pineau T., Hilbert D.M., McPhail T., Lee S.S., Kimura S., Nebert D.W., Rudikoff S., Ward J.M., Gonzalez F.J. (1995). Immune system impairment and hepatic fibrosis in mice lacking the dioxin-binding Ah receptor. Science.

[48] Baba T., Mimura J., Nakamura N., Harada N., Yamamoto M., Morohashi K., Fujii-Kuriyama Y. (2005). Intrinsic function of the aryl hydrocarbon (dioxin) receptor as a key factor in female reproduction. Mol. Cell. Biol..

[49] Andersson P., McGuire J., Rubio C., Gradin K., Whitelaw M.L., Pettersson S., Hanberg A., Poellinger L. (2002). A constitutively active dioxin/aryl hydrocarbon receptor induces stomach tumors. Proc. Natl. Acad. Sci. USA.

[50] Murray I.A., Patterson A.D., Perdew G.H. (2014). Aryl hydrocarbon receptor ligands in cancer: Friend and foe. Nat. Rev. Cancer.

[51] Zelante T., Iannitti R.G., Cunha C., De Luca A., Giovannini G., Pieraccini G., Zecchi R., D’Angelo C., Massi-Benedetti C., Fallarino F. (2013). Tryptophan catabolites from microbiota engage aryl hydrocarbon receptor and balance mucosal reactivity via interleukin-22. Immunity.

[52] Opitz C.A., Litzenburger U.M., Sahm F., Ott M., Tritschler I., Trump S., Schumacher T., Jestaedt L., Schrenk D., Weller M. (2011). An endogenous tumour-promoting ligand of the human aryl hydrocarbon receptor. Nature.

[53] Novikov O., Wang Z., Stanford E.A., Parks A.J., Ramirez-Cardenas A., Landesman E., Laklouk I., Sarita-Reyes C., Gusenleitner D., Li A. (2016). An aryl hydrocarbon receptor-mediated amplification loop that enforces cell migration in ER-/PR-/Her2-human breast cancer cells. Mol. Pharmacol..

[54] Stanford E.A., Ramirez-Cardenas A., Wang Z., Novikov O., Alamoud K., Koutrakis P., Mizgerd J.P., Genco C.A., Kukuruzinska M., Monti S. (2016). Role for the aryl hydrocarbon receptor and diverse ligands in oral squamous cell carcinoma migration and tumorigenesis. Mol. Cancer Res..

[55] DiNatale B.C., Smith K., John K., Krishnegowda G., Amin S.G., Perdew G.H. (2012). Ah receptor antagonism represses head and neck tumor cell aggressive phenotype. Mol. Cancer Res..

[56] John K., Lahoti T.S., Wagner K., Hughes J.M., Perdew G.H. (2014). The Ah receptor regulates growth factor expression in head and neck squamous cell carcinoma cell lines. Mol. Carcinog..

[57] Flaveny C.A., Murray I.A., Chiaro C.R., Perdew G.H. (2009). Ligand selectivity and gene regulation by the human aryl hydrocarbon receptor in transgenic mice. Mol. Pharmacol..

[58] Hubbard T.D., Murray I.A., Bisson W.H., Sullivan A.P., Sebastian A., Perry G.H., Jablonski N.G., Perdew G.H. (2016). Divergent Ah receptor ligand selectivity during hominin evolution. Mol. Biol. Evol..

[59] Faber S.C., Soshilov A.A., Giani Tagliabue S., Bonati L., Denison M.S. (2018). Comparative In Vitro and In Silico Analysis of the Selectivity of Indirubin as a Human Ah Receptor Agonist. Int. J. Mol. Sci..

[60] González-Sarrías A., Espín J.-C., Tomás-Barberán F.A., García-Conesa M.-T. (2009). Gene expression, cell cycle arrest and MAPK signalling regulation in Caco-2 cells exposed to ellagic acid and its metabolites, urolithins. Mol. Nutr. Food Res..

[61] Sharma M., Li L., Celver J., Killian C., Kovoor A., Seeram N.P. (2010). Effects of fruit ellagitannin extracts, ellagic acid, and their colonic metabolite, Urolithin A, on Wnt signaling. J. Agric. Food Chem..

[62] González-Sarrías A., Núñez-Sánchez M.Á., Tomé-Carneiro J., Tomás-Barberán F.A., García-Conesa M.T., Espín J.C. (2016). Comprehensive characterization of the effects of ellagic acid and urolithins on colorectal cancer and key-associated molecular hallmarks: MicroRNA cell specific induction of CDKN1A (p21) as a common mechanism involved. Mol. Nutr. Food Res..

[63] Kasimsetty S.G., Bialonska D., Reddy M.K., Ma G., Khan S.I., Ferreira D. (2010). Colon cancer chemopreventive activities of pomegranate ellagitannins and urolithins. J. Agric. Food Chem..

[64] Nuñez-Sánchez M.A., Dávalos A., González-Sarrías A., Casas-Agustench P., Visioli F., Monedero-Saiz T., García-Talavera N.V., Gómez-Sánchez M.B., Sánchez-Álvarez C., García-Albert A.M. (2015). MicroRNAs expression in normal and malignant colon tissues as biomarkers of colorectal cancer and in response to pomegranate extracts consumption: Critical issues to discern between modulatory effects and potential artefacts. Mol. Nutr. Food Res..

[65] Totiger T.M., Srinivasan S., Jala V.R., Lamichhane P., Dosch A.R., Gaidarski A.A., Joshi C., Rangappa S., Castellanos J., Vemula P.K. (2018). Urolithin A, a novel natural compound to target PI3K/AKT/mTOR pathway in pancreatic cancer. Mol. Cancer Ther..

[66] Dahiya N.R., Chandrasekaran B., Kolluru V., Ankem M., Damodaran C., Vadhanam M.V. (2018). A natural molecule, urolithin A, downregulates androgen receptor activation and suppresses growth of prostate cancer. Mol. Carcinog..

[67] García-Villalba R., Espín J.C., Tomás-Barberán F.A. (2016). Chromatographic and spectroscopic characterization of urolithins for their determination in biological samples after the intake of foods containing ellagitannins and ellagic acid. J. Chromatogr. A.

[68] Long W.P., Pray-Grant M., Tsai J.C., Perdew G.H. (1998). Protein kinase C activity is required for aryl hydrocarbon receptor pathway-mediated signal transduction. Mol. Pharmacol..

[69] Singh S.S., Hord N.G., Perdew G.H. (1996). Characterization of the activated form of the aryl hydrocarbon receptor in the nucleus of HeLa cells in the absence of exogenous ligand. Arch. Biochem. Biophys..

