# Supplementary material for: Urolithin A Is a Dietary Microbiota-Derived Human Aryl Hydrocarbon Receptor Antagonist
Source: Metabolites. 2018 Nov 29;8(4):86. doi: 10.3390/metabo8040086 (PMC6315438; doi:10.3390/metabo8040086)
Supplement: Supplementary file 1 [file metabolites-08-00086-s001.pdf]

SUPPLEMENTARY MATERIAL

Urolithin A Is a Dietary Microbiota-Derived Human Aryl Hydrocarbon Receptor Antagonist

Gulsum E. Muku [1,2], Iain A. Murray [1], Juan C. Espín [3] and Gary H. Perdew [1,*]

[1] Center for Molecular Toxicology and Carcinogenesis, Department of Veterinary and Biomedical Sciences, The Pennsylvania State University, University Park, PA 16802, USA; gem186@psu.edu (G.E.M.); iam1@psu.edu (I.A.M.)
[2] The Huck Institutes of the Life Sciences, The Pennsylvania State University, University Park, PA 16802, USA
[3] Laboratory of Food & Health, Research Group on Quality, Safety and Bioactivity of Plant Foods, Department of Food Science and Technology, CEBAS-CSIC, 30100 Murcia, Spain; jcespin@cebas.csic.es
* Correspondence: ghp2@psu.edu; Tel.: +1-814-865-0400

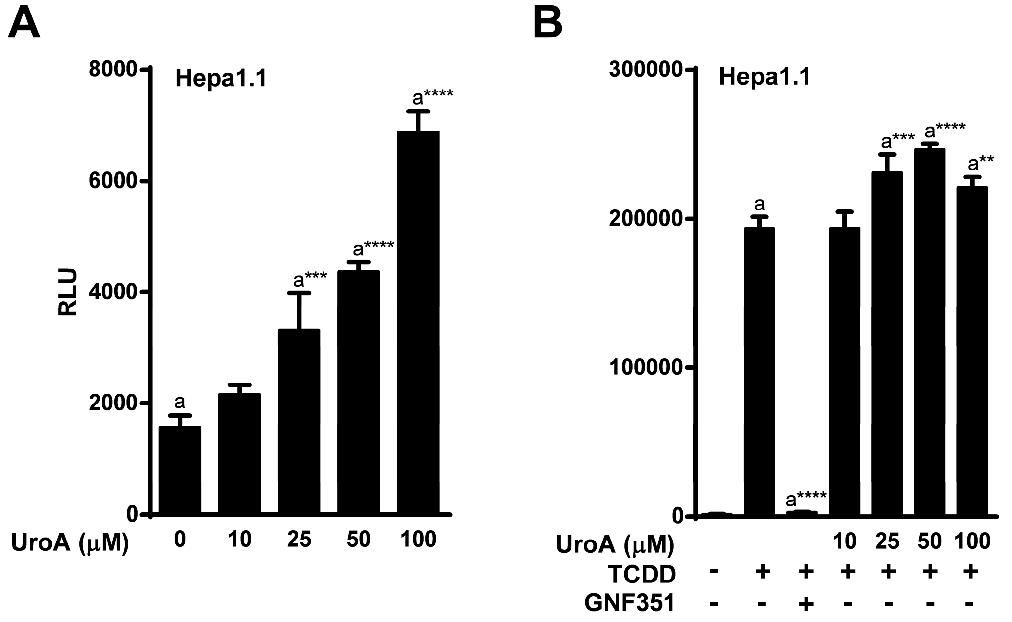

Figure S1. The agonist and antagonist activity of UroA was assessed in Hepa1.1 at higher doses. (A) Hepa1.1 cells were treated with 10, 25, 50, 100 μM of UroA in the absence of exogenous ligands or (B) in the presence of TCDD for 4 h.

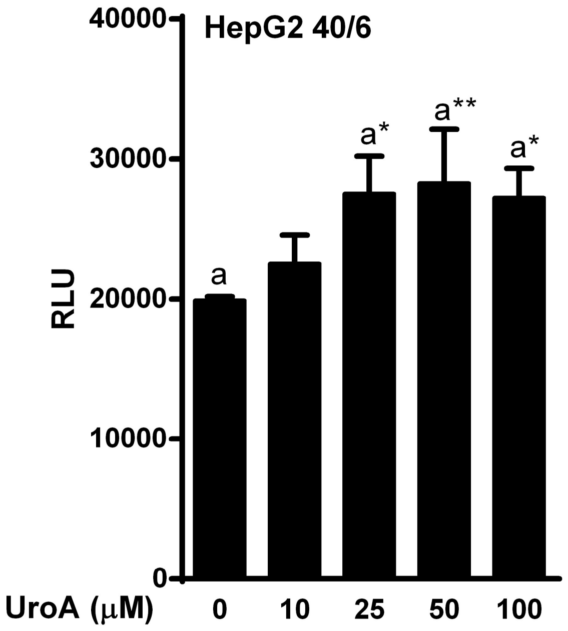

Figure S2. The agonist activity of UroA was assessed in HepG2 40/6 at higher doses. (A) HepG2 40/6 cells were treated with 10, 25, 50, 100 μM of UroA in the absence of exogenous ligands for 4 h.

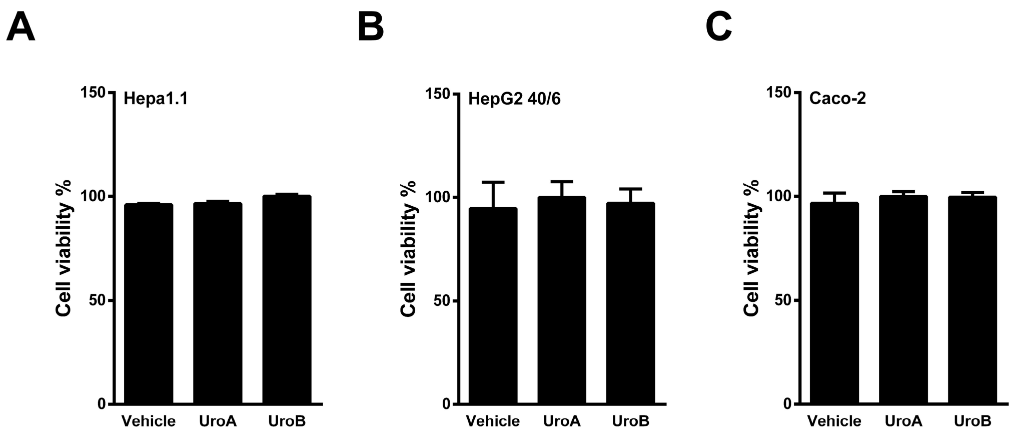

Figure S3. UroA does not cause cytotoxicity in Hepa1.1, HepG2 (40/6) or Caco-2 cells. MTT assay was performed on (a) Hepa1.1 or HepG2 (40/6) cells treated with 10 μM of UroA or B for 4 h, (b) Caco-2 cells treated with 10 μM of UroA for 16 h.
